# Activation of G Proteins by Guanine Nucleotide Exchange Factors Relies on GTPase Activity

**DOI:** 10.1371/journal.pone.0151861

**Published:** 2016-03-17

**Authors:** Rob J. Stanley, Geraint M. H. Thomas

**Affiliations:** 1 CoMPLEX, University College London, London, United Kingdom; 2 Department of Cell & Developmental Biology, University College London, London, United Kingdom; BioScience Project, UNITED STATES

## Abstract

G proteins are an important family of signalling molecules controlled by guanine nucleotide exchange and GTPase activity in what is commonly called an ‘activation/inactivation cycle’. The molecular mechanism by which guanine nucleotide exchange factors (GEFs) catalyse the activation of monomeric G proteins is well-established, however the complete reversibility of this mechanism is often overlooked. Here, we use a theoretical approach to prove that GEFs are unable to positively control G protein systems at steady-state in the absence of GTPase activity. Instead, positive regulation of G proteins must be seen as a product of the competition between guanine nucleotide exchange and GTPase activity—emphasising a central role for GTPase activity beyond merely signal termination. We conclude that a more accurate description of the regulation of G proteins via these processes is as a ‘balance/imbalance’ mechanism. This result has implications for the understanding of intracellular signalling processes, and for experimental strategies that rely on modulating G protein systems.

## Introduction

G proteins are an important and universal family of intracellular signalling molecules, incorporating both the alpha subunits of heterotrimeric G proteins and the Ras small monomeric G proteins. Most G proteins bind guanine nucleotides (GDP, GTP) in a strongly conserved nucleotide binding pocket—an ancient mechanism preserved in both eukaryotes and prokaryotes [1–3]. Typically, G proteins transition between two discrete conformations with distinct signalling functions depending on which nucleotide is bound, and so G proteins are often referred to as ‘molecular switches’. G protein regulatory systems are crucial components of many intracellular processes and incorrect regulation of G proteins has been implicated in disease: cancer [4–6], cardiovascular disease [7], genetic disorders [8], among many others.

**Fig 1:**
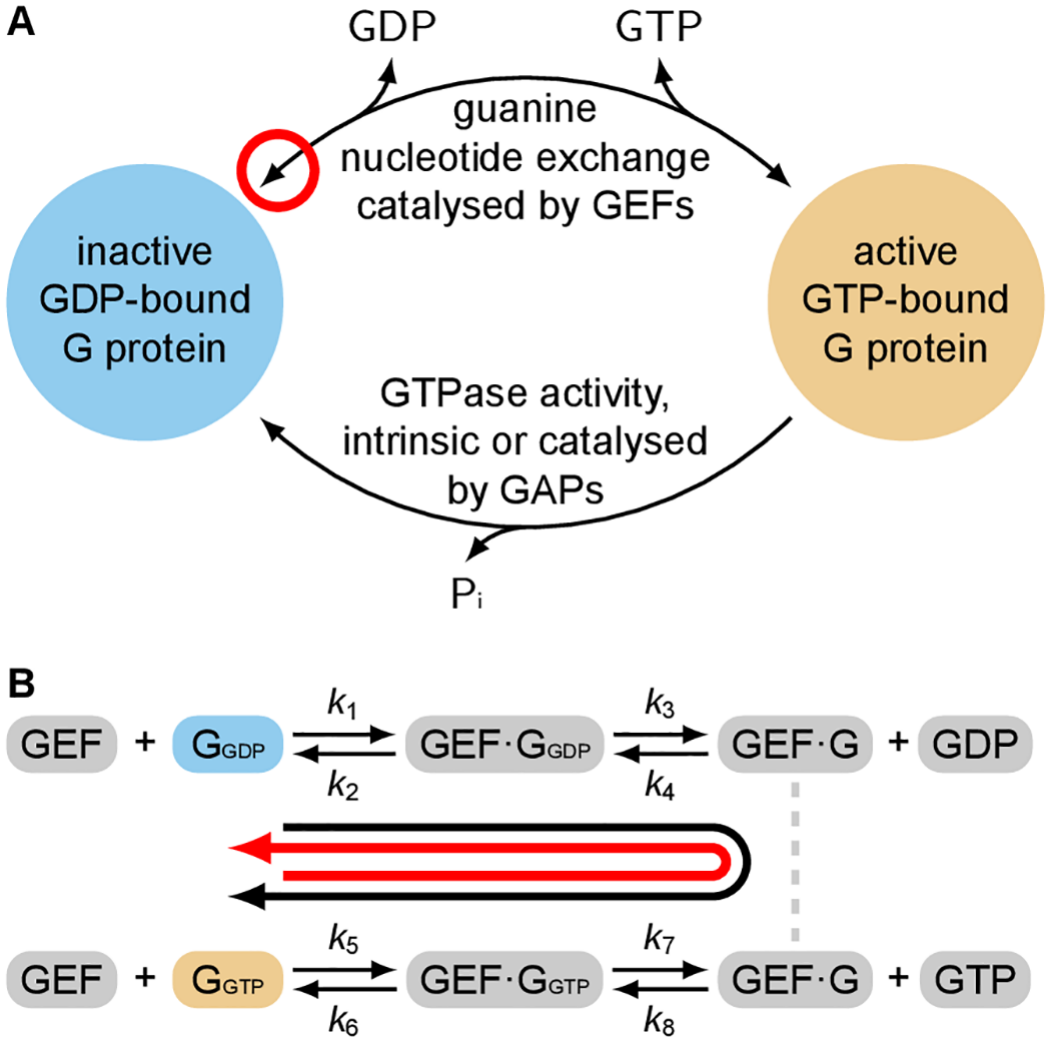
The activation of G proteins is regulated by GEFs and GTPase activity. **A** G proteins are controlled by GEFs which catalyse the sequential release and binding of guanine nucleotides, and by GTPase activity (both intrinsic and GAP-mediated) which hydrolyses bound GTP to form GDP and Pi. The red circle highlights that the GEF mechanism is completely reversible. **B** The reversible mechanism by which a GEF catalyses guanine nucleotide exchange on a G protein proceeds through a series of GEF·G protein complexes [13]. Parameters *k*_*i*_ are kinetic rates which are unique to each G protein:GEF system. Associated species (free GEF, GTP, GDP) have not been drawn. The thick black arrow identifies forwards nucleotide exchange, catalysing the activation of the G protein. The thick red arrow identifies reverse nucleotide exchange, catalysing the inactivation of the G protein.

Regulation of G protein activation state is largely controlled by two mechanisms (Fig 1A) and is commonly described as an ‘activation/inactivation cycle’ between the GTP-bound ‘on/active’ state and the GDP-bound ‘off/inactive’ state [9, 10]. Activation of G proteins is enabled by accessory proteins which catalyse guanine nucleotide exchange—the sequential release of GDP and binding of GTP. For monomeric G proteins these are known as guanine nucleotide exchange factors (GEFs). For heterotrimeric G proteins, G protein coupled receptors (GPCRs) fulfil this role. Inactivation of G proteins is controlled by GTPase activity which may either be intrinsic, or be provided via accessory GTPase-activating proteins (GAPs). It is generally thought that GTPase activity is required for the termination of G protein signalling but that it is not essential for signal transmission [11].

An often overlooked property of GEFs is that their catalytic mechanism is completely reversible (Fig 1B) [12]. GEF-binding is not specific to GDP-bound G protein—GEFs can also bind to GTP-bound G protein and catalyse the reverse nucleotide exchange, GTP to GDP. In this way GEFs are capable of inactivating G proteins [13]. The extent to which the reversibility of this mechanism has been overlooked is demonstrated by the sheer number of publications which include diagrams where arrows corresponding to GEF-mediated regulation are drawn as unidirectional—missing the reverse arrowhead highlighted in Fig 1A. This error is perhaps best illustrated by its occurrence in core biology textbooks, for example:

- Figures 3–66 and 3–68 in [14]
- Figures 16–15 and 16–16 in [15]
- Figure 4, box 12–2 in [16]
- Figure 13.40 in [17]
- Figure 19–40 in [18]
- Figure 7.12A in [19]
- Figure 10.3 and 10.4 in [20]
- Figure 42.4 in [21]

There has been recent renewed interest in understanding the roles and functions of GEFs based on a proper consideration of their thermodynamics and enzyme kinetics [12, 22, 23]. Additionally, G protein:GEF interactions have previously been thoroughly studied in the context of thermodynamics [24]. Here we develop the existing theoretical understanding of G protein regulation by GEFs and GTPase activity through further exploring the consequences of the reversibility of the GEF mechanism. We use mathematical methods to investigate a set of generic and minimal G protein regulatory systems independent of measured kinetic rates, in the context of the physiologically important steady-state dynamics. This allows us to comment and draw conclusions on the qualitative behaviours of similar G protein:GEF:GTPase systems under a wide variety of conditions. These minimal systems consist of a single G protein, a single GEF, and either intrinsic or GAP-mediated GTPase activity, with no external influences or localisation effects, and under the assumption that the system is homogeneous in space. For illustrative purposes, to ensure that the included figures are physiologically plausible, our simulations use parameters described for the Ran:RCC1:RanGAP1 system [25, 26]—chosen as this provides a complete set of rate constants for the G protein:GEF interaction (as described in Fig 1B) and corresponding Michaelis-Menten constants for the G protein:GAP interaction.

## Results

### Qualitative differences between reversible and irreversible mechanisms

To demonstrate the qualitative difference between a reversible and an irreversible mechanism we derived mass-action models of the GEF mechanism (Fig 1B, Methods) and an artificial irreversible mechanism generated by disallowing release of GTP from the GEF·G protein complex.

**Fig 2:**
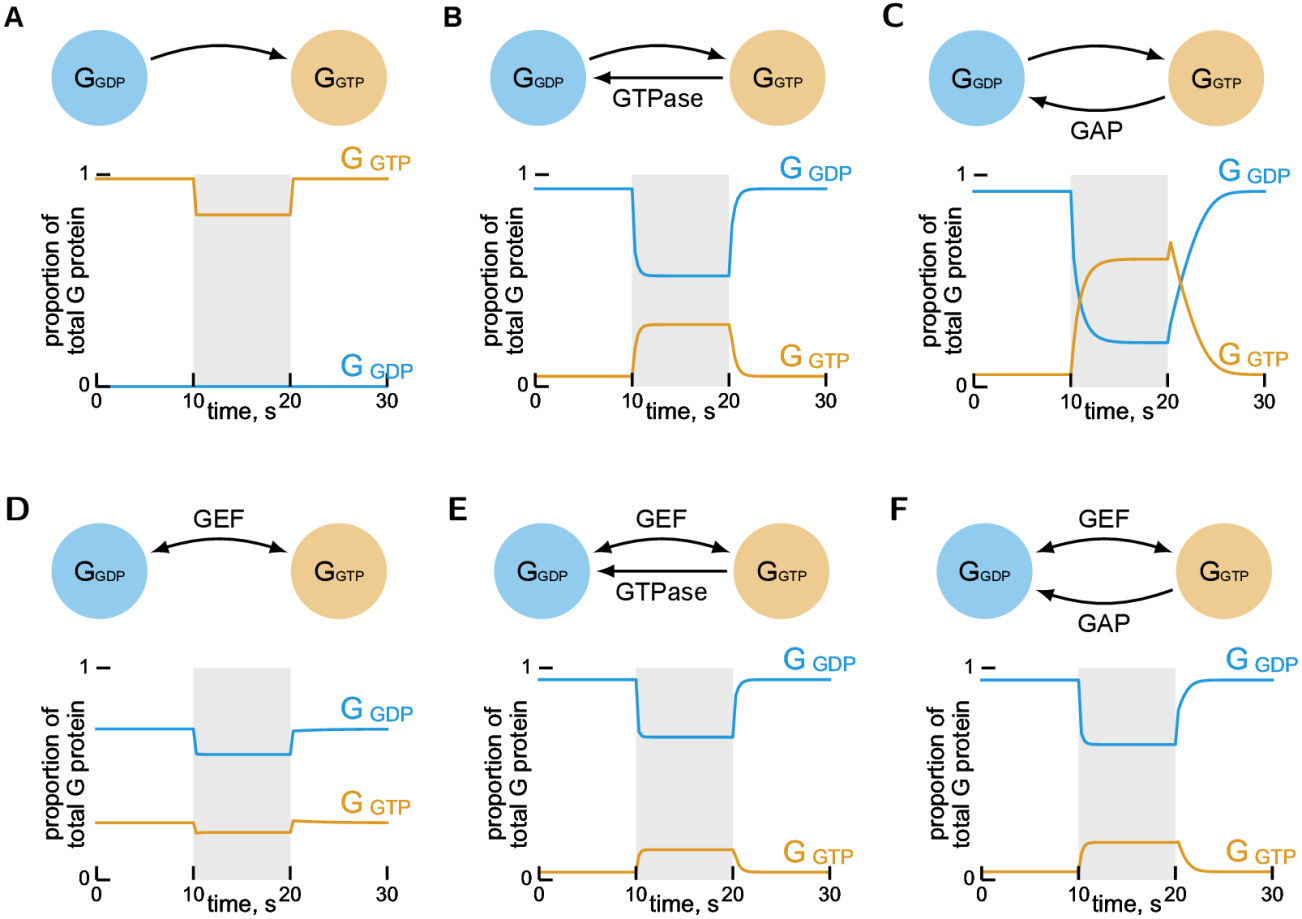
Apparent activation of G proteins via GEFs is only observed when GTPase activity is present. Simulation of mass-action models, see Methods. *G*_GXP_ denotes GXP-bound G protein. The shaded region denotes stimulation of the system by increasing the active GEF 10-fold from its basal concentration. For all simulations, steady-state concentrations were used as the initial conditions. Mass corresponding to GEF·G protein complexes has not been drawn. **A, B, C** An artificial irreversible model, constructed by assuming the rate of release of GTP from the active GEF·G protein complex is zero. **D, E, F** The reversible GEF mechanism (Fig 1B).

The reversible and irreversible models were simulated: in the absence of GTPase activity (Fig 2A and 2D); with intrinsic GTPase activity, modelled by exponential decay (Fig 2B and 2E); and with GAP-mediated GTPase activity, modelled using the Michaelis-Menten equation (Fig 2C and 2F).

In the presence of either form of GTPase activity both reversible and irreversible mechanisms display qualitatively similar behaviour (ignoring differences in magnitude) which is consistent with observations of GEF-mediated activation of G proteins in a wide range of biological systems [27–31].

In the absence of GTPase activity we see a qualitative difference in the behaviour of the two mechanisms—each distinct from their shared behaviour in the presence of GTPase activity. While both mechanisms show an inhibitory effect (which will be discussed below in more detail for the GEF mechanism), the steady-state concentrations of active and inactive G protein differ substantially. This demonstrates how the assumption of an irreversible model could possibly lead to incorrect conclusions, at least when considering extremal (i.e. diseased) states.

### GEFs act to attain a constant ratio of inactive to active G protein

We derived a simplified quasi-steady-state model of the GEF mechanism (Fig 1B) in an equivalent manner to the derivation of the Michaelis-Menten equation [32–35]. This quasi-steady-state model captures the behaviour of a generic and minimal G protein regulatory system in a single equation:

$$\frac{d[G_{\text{GTP}}]}{dt}=\frac{k_{\text{fwd}}\left(\left[G_{\text{GDP}}\right]-\kappa \left[G_{\text{GTP}}\right]\right)e_{0}}{K_{0}+K_{1}\left[G_{\text{GDP}}\right]+K_{2}\left[G_{\text{GTP}}\right]}-f_{\text{GTPase}}$$

Here [*G*_GXP_] is the concentration of GXP-bound G protein and *κ* is the ratio of the backwards to the forwards kinetic rates. (For definitions of the other parameters see the Methods section.)

At steady-state (setting the above equation equal to zero), in the absence of GTPase activity, we find that the ratio of inactive to active G protein must always equal the value of the constant *κ*. An equivalent statement is: GEFs act to produce a constant proportion of active G protein. While the ratio of inactive to active G protein (*κ*) and proportion of active G protein ($\frac{1}{\kappa +1}$) will vary for different G protein:GEF systems, these values will remain constant within a system, independent of the G protein or GEF concentrations.

### GEFs can be inhibitory

The commonly used description of GEFs as ‘activators’ of G proteins is contradicted by the inhibitory effect seen when the GEF mechanism is simulated in the absence of GTPase activity (Fig 2D).

**Fig 3:**
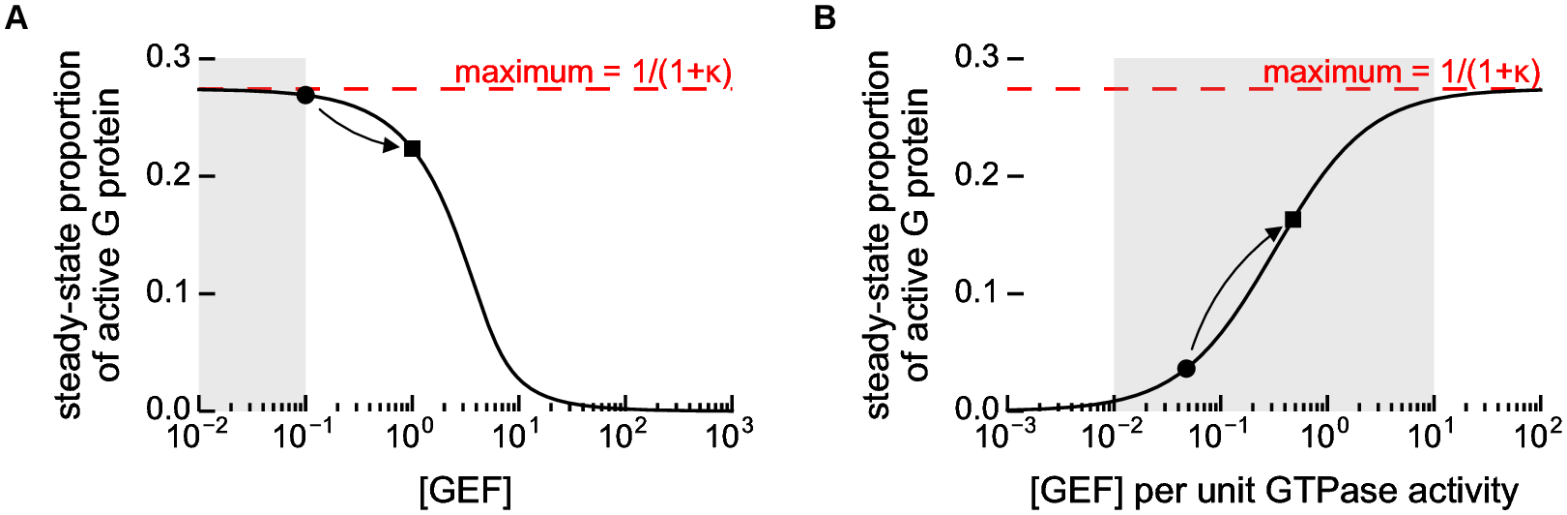
GTPase activity restores the ability of GEFs to positively regulate a G protein by moving the system away from equilibrium. The relationship between the concentration of GEF and the steady-state proportion of active G protein. The concentration of active G protein cannot increase above a theoretical maximum proportion derived from the ratio of the total backwards and forwards catalytic rates of the GEF (*κ*). The markers and arrow demonstrate the effect seen before (circle) and during (square) the stimulation shown in Fig 2D and 2E. The shaded region denotes the region which is most likely to be physiologically relevant. **A** In the absence of GTPase activity, Eq 5, increasing the GEF concentration can only decrease the steady-state concentration of active G protein, instead producing GEF·G protein complexes. **B** In the presence of GTPase activity, Eq 7, the steady-state concentration of active G protein is suppressed. Increasing the (relative) concentration of GEF acts to counter this suppression, driving the activation state back towards the maximum.

The inhibitory effect can be explained by an equivalent increase in the concentrations of intermediate GEF·G protein complexes. Values for the concentrations of these intermediate complexes were derived as part of the construction of the quasi-steady-state model. Using these values, we obtained an equation for the proportion of (free) active G protein in terms of the total concentration of GEF. This equation is plotted in Fig 3A. Mathematically, we are then able to prove that in the absence of GTPase activity the concentration of active G protein is inversely related to the total concentration of GEF. As the concentration of GEF increases, the concentration of active G protein will always decrease, and vice-versa.

Note that a high concentration of GEF will also lead to a faster total catalytic rate (a larger *V*_max_). This suggests that there will be a tradeoff in terms of increasing the concentration of GEF: a low concentration of GEF means that there will be little inhibition, but a slow total rate; a high concentration of GEF will lead to inhibition, but a fast total rate. We therefore hypothesise that for a healthy G protein system, the concentration of GEF will lie in a physiologically relevant region, where the inhibitory effect is not so pronounced, but where there is still sufficient GEF to catalyse nucleotide exchange at an appropriate rate.

### GTPase activity has a functional role in the observed activation of G proteins

The simulations of the GEF mechanism show that inclusion of GTPase activity is sufficient to restore an apparent GEF-mediated activation (Fig 2E and 2F). By comparing these with the simulation of the system without GTPase activity (Fig 2D) we can see how this activation arises. Initially, due to the GTPase activity, the activation state reached by the system is suppressed—it is much reduced from the maximum proportion of active G protein achieved in the absence of GTPase activity. An increase in the concentration of GEF is then able to positively regulate the system by moving the activation state closer to that maximum. For intrinsic GTPase activity we obtained an equation which describes the effect of the relative rates of GEF-catalysed nucleotide exchange and GTPase activity on the proportion of G protein which is active. This equation is plotted in Fig 3B, where we see a sigmoidal response—increasing the concentration of GEF (relative to the GTPase activity) increases the concentration of active G protein. Again this allows us to hypothesise that, for a healthy G protein system, the relative rates of nucleotide exchange and GTPase activity must lie in this sigmoidal region, in order for the system to properly respond to an activating or inhibitory signal.

Together, this demonstrates a requirement for GTPase activity for the observable activation of G proteins by GEFs, at least within a minimal G protein regulatory system. The proposed mechanism of regulation for such a G protein:GEF:GTPase system can be summarised as follows: 1. GTPase activity inactivates the G protein system by altering the ratio of inactive to active G protein away from a GEF-mediated equilibrium. 2. If the rate of guanine nucleotide exchange increases, or the GTPase activity decreases, the proportion of active G protein will then move towards the GEF-mediated equilibrium, activating the system. We note that our result can be described in lay terms as: ‘G proteins must be first turned off, before they can be turned on’.

## Discussion

We have shown that there are certain properties of GEF-mediated regulation of G proteins that arise from the reversibility of its mechanism and which are independent of specific kinetic rates. The complete reversibility of the GEF mechanism means that at steady-state any GEF acts to produce a constant ratio of inactive to active G protein—giving a theoretical maximum proportion of active G protein. Once this maximum is attained, then any subsequent increase in the concentration of GEF—the ‘activator’ of the system—cannot increase the concentration of active G protein. Instead we will observe inhibition caused by creation of excess intermediate GEF·G protein complexes.

We urge caution against the naïve description of GEFs as ‘enzymes that activate G proteins’ and against representations that show this mechanism as irreversible, as we have shown how these shorthands can distort our understanding of the underlying biology. We would instead suggest that GEFs are thought of as enzymes that act to attain an equilibrium—a balance—of active and inactive G protein—a definition which is entirely consistent with their existing designation as ‘exchange factors’. Correspondingly, we see GTPase activity as having two main roles: to drive the system away from this equilibrium—to create an imbalance—and so permit positive regulation by GEFs; and to confer a unique directionality on the G protein regulatory ‘cycle’. Therefore we propose that G protein signalling, at least in its minimal form discussed here, is better described as operating through ‘regulated balance/imbalance’.

Both the complete reversibility of guanine nucleotide exchange and associated requirement for GTPase activity as a functional component in the activation of G proteins has previously been under-appreciated. This may be due to the almost exclusive use of experimental systems where the GDP form of the G protein is the unique starting condition and where uptake of GTP is monitored as the GEF assay. We also note that our simulations show that an artificial irreversible mechanism (Fig 2B and 2C) and reversible GEF mechanism (Fig 2E and 2F) have similar profiles in the presence of GTPase activity and so under many conditions it may be difficult to experimentally distinguish these mechanisms.

We predict that experimental protocols which attempt to regulate G proteins by the over-expression of a GEF are likely to produce unexpected behaviour. We expect that in many cases this may cause inhibition of the G protein rather than activation (Fig 3A). Activation of G proteins should therefore be preferentially targeted by reduction of the relevant GTPase activity (Fig 3B). Note that these results remain consistent with the long-established use of dominant negative mutants for the inhibition of G protein systems [36, 37]. We accept that many previous studies that have ignored the reversibility of GEFs will have made conclusions that are valid under many conditions. But we stress that in extremal scenarios (such as in disease) those conclusions may not always hold.

Additionally, we hope that this new perspective in considering the control of G proteins will lead to novel approaches for the control of G protein systems. GEFs have previously been suggested as potential therapeutic targets [13]. Our results extend this to a novel, and seemingly paradoxical, mechanism by which over-expression of an activator could lead to the inhibition of its substrate. This may have implications in G protein systems with diminished GTPase activity, for example constitutively active transforming mutations in Ras common in cancers [38], where additional GAP activity would have no effect but where sequestration of active G protein by a GEF may be useful alternative.

It is important to note that our mathematical results explicitly apply to the system we have considered: a homogeneous system with deterministic kinetics with no localisation or other external interactions, and under the reasonable assumption that the majority of its functional signalling is due to the steady-state behaviour. The precise equilibrium ratios, total rates, and inhibitory effects for any system that follows these requirements will depend on the specific kinetic rates for the GEF and the strength of GTPase activity, but the overall qualitative characteristics should remain consistent. Conclusions based on alternative mechanisms would require further analysis, for instance systems with an implicit G protein·GEF·GAP complex [39], or systems with changes in localisation, for example reversible membrane-tethering of Arf during activation [40]. While we certainly accept that some additional complications may alter the behaviour of specific G protein:GEF:GTPase systems, we suggest that, rather than speculation, these modified systems have their own behaviours analysed mathematically.

## Methods

The following mathematical analysis uses the notation:

- G protein without nucleotide bound → *G*
- G protein with GDP bound → *G*_GDP_
- G protein with GTP bound → *G*_GTP_
- GEF → *E*

The volume concentration of a species *S* will be denoted by [*S*].

### Mass-action model

A deterministic ordinary differential equation (ODE) model of the GEF mechanism (Fig 1B) was derived using the law of mass-action:

$$\begin{array}{ll} \frac{\text{d}[E]}{\text{d}t} & =-[E](k_{1}[G_{\text{GDP}}]+k_{5}[G_{\text{GTP}}])+k_{2}[E\cdot G_{\text{GDP}}]+k_{6}[E\cdot G_{\text{GTP}}]\\ \frac{\text{d}[E\cdot G_{\text{GDP}}]}{\text{d}t} & =-(k_{2}+k_{3})[E\cdot G_{\text{GDP}}]+k_{1}[G_{\text{GDP}}][E]+k_{4}[E\cdot G][\text{GDP}]\\ \frac{\text{d}[E\cdot G_{\text{GTP}}]}{\text{d}t} & =-(k_{6}+k_{7})[E\cdot G_{\text{GTP}}]+k_{5}[G_{\text{GTP}}][E]+k_{8}[E\cdot G][\text{GTP}]\\ \frac{\text{d}[E\cdot G]}{\text{d}t} & =-(k_{4}[\text{GDP}]+k_{8}[\text{GTP}])[E\cdot G]+k_{3}[E\cdot G_{\text{GDP}}]+k_{7}[E\cdot G_{\text{GTP}}]\\ \frac{\text{d}[G_{\text{GDP}}]}{\text{d}t} & =-k_{1}[E][G_{\text{GDP}}]+k_{2}[E\cdot G_{\text{GDP}}]+f_{\text{GTPase}}\\ \frac{\text{d}[G_{\text{GTP}}]}{\text{d}t} & =-k_{5}[E][G_{\text{GTP}}]+k_{6}[E\cdot G_{\text{GTP}}]-f_{\text{GTPase}} \end{array}$$

We assume: for systems with no GTPase activity, *f*_GTPase_ = 0; for systems with intrinsic GTPase activity, *f*_GTPase_ = *k*_ase_[*G*_GTP_]; and for systems with GAP-mediated GTPase activity, $f_{\text{GTPase}}=\frac{k_{\text{ase}}\left[G_{\text{GTP}}\right]f_{0}}{K_{m}+\left[G_{\text{GTP}}\right]}$ where *f*_0_ is the total concentration of GAP.

There is an equation for the conservation of mass of GEF:

$$e_{0}=\left[E\right]+\left[E\cdot G_{\text{GDP}}\right]+\left[E\cdot G_{\text{GTP}}\right]+\left[E\cdot G\right]$$ (1)

And an equation for the conservation of mass of G protein:

$$g_{0}=\left[G_{\text{GDP}}\right]+\left[G_{\text{GTP}}\right]+\left[E\cdot G_{\text{GDP}}\right]+\left[E\cdot G_{\text{GTP}}\right]+\left[E\cdot G\right]$$ (2)

### Simulation of the mass-action model

The parameters used for the simulations in Fig 2 are summarised in S1 Table. For G protein:GEF interactions, parameters measured for the Ran:RCC1:RanGAP1 system were used [25, 26]; concentrations of Ran and RCC1 were estimated from the PaxDB database [41] scaled by an estimate of the total concentration of proteins given by [42]; concentrations of nucleotides were taken from [43]. The irreversible model was generated by setting *k*_7_ = 0. (Alternative irreversible models could be generated by setting any one or more of the reverse reaction rates to zero.)

All simulations were started from steady-state and generated by (deterministic) numerical integration of the mass-action equations, with the exception of free enzyme concentration [*E*] which was calculated from the total mass of enzyme, Eq 1, with:

- *e*_0_ = 0.1 during 0 ≤ *t* < 10
- *e*_0_ = 1.0 during 10 ≤ *t* < 20
- and free GEF (*E*) removed from the simulation until *e*_0_ = 0.1 during *t* ≥ 20.

The complete implementation can be found in S1 Simulations.

### Quasi-steady-state model

Quasi-steady-state solutions for the intermediate enzyme complexes of the GEF mechanism (Fig 1B) were derived using the framework of [35] (S1 Fig):

$$\begin{array}{ll} \left[E\right] & =\left(\frac{K_{0}}{K_{0}+K_{1}[G_{\text{GDP}}]+K_{2}[G_{\text{GTP}}]}\right)e_{0}\\ \left[E\cdot G_{\text{GDP}}\right] & =\left(\frac{K_{1}^{d}[G_{\text{GDP}}]+K_{2}^{d}[G_{\text{GTP}}]}{K_{0}+K_{1}[G_{\text{GDP}}]+K_{2}[G_{\text{GTP}}]}\right)e_{0}\\ \left[E\cdot G_{\text{GTP}}\right] & =\left(\frac{K_{1}^{t}[G_{\text{GDP}}]+K_{2}^{t}[G_{\text{GTP}}]}{K_{0}+K_{1}[G_{\text{GDP}}]+K_{2}[G_{\text{GTP}}]}\right)e_{0}\\ \left[E\cdot G\right] & =\left(\frac{K_{1}^{g}[G_{\text{GDP}}]+K_{2}^{g}[G_{\text{GTP}}]}{K_{0}+K_{1}[G_{\text{GDP}}]+K_{2}[G_{\text{GTP}}]}\right)e_{0} \end{array}$$

where the $K_{i}^{x}$ and the *K*_*i*_ are summary parameters (defined in S1 Table).

These quasi-steady-state solutions were substituted into the equation for the rate of change of [*G*_GTP_] given in the mass-action model to obtain a quasi-steady-state model for a generic GEF acting on a generic G protein:

$$\frac{d[G_{\text{GTP}}]}{dt}=\frac{k_{\text{fwd}}\left(\left[G_{\text{GDP}}\right]-\kappa \left[G_{\text{GTP}}\right]\right)e_{0}}{K_{0}+K_{1}\left[G_{\text{GDP}}\right]+K_{2}\left[G_{\text{GTP}}\right]}-f_{\text{GTPase}}$$ (3)

where *k*_fwd_ is the product of the forward kinetic rates, and *κ* is the ratio of the backwards to the forwards kinetic rates, multiplied by the ratio of GDP to GTP.

This equation does not consider mass held in GEF·G protein intermediate complexes and so is only a good approximation when *e*_0_ ≪ *g*_0_. Note that with *f*_GTPase_ = 0 this model reduces to the Michaelis-Menten equation when *y* = 0, and is equivalent to the equation used by [23] when the concentration of GTP is absorbed into the summary parameters.

### Steady-state ratio of inactive to active G protein

At steady-state with *f*_GTPase_ = 0, Eq 3 implies:

$$\left[G_{\text{GDP}}\right]=\kappa \left[G_{\text{GTP}}\right]$$ (4)

Assuming that *e*_0_ ≪ *g*_0_, Eq 2 simplifies to *g*_0_ = [*G*_GDP_] + [*G*_GTP_], into which Eq 4 can be substituted to obtain:

$$\frac{\left[G_{\text{GTP}}\right]}{g_{0}}=\frac{1}{\kappa +1}$$

This is the maximum steady-state proportion of active G protein.

### Active G protein as a function of GEF concentration (without GTPase activity)

The effect of increasing the concentration of GEF on the steady-state concentration of active G protein in the absence of GTPase activity (*f*_GTPase_ = 0) was investigated.

The quasi-steady-state solutions for the intermediate enzyme complexes and Eq 4 were substituted into Eq 2 to obtain:

$$0=\left(\kappa +1\right){\left[G_{\text{GTP}}\right]}^{2}+2b\left[G_{\text{GTP}}\right]-K_{s}g_{0}$$

where $b=\frac{1}{2}(e_{0}-g_{0}+\left(\kappa +1\right)K_{s})$ and $K_{s}=\frac{K_{0}}{\left(K_{1}\kappa +K_{2}\right)}$.

This quadratic equation has one positive solution:

$$\left[G_{\text{GTP}}\right]=\frac{1}{\kappa +1}\left(-b+\sqrt{b^{2}+\left(\kappa +1\right)K_{s}g_{0}}\right)$$

Alternatively, the proportion of active G protein is:

$$\frac{\left[G_{\text{GTP}}\right]}{g_{0}}=\frac{1}{g_{0}\left(\kappa +1\right)}\left(-b+\sqrt{b^{2}+\left(\kappa +1\right)K_{s}g_{0}}\right)$$ (5)

We are interested in the rate of change of [*G*_GTP_] with respect to *e*_0_, the total concentration of GEF. As *b* (and only *b*) is a function of *e*_0_, we can examine:

$$\frac{d[G_{\text{GTP}}]}{db}=\frac{1}{\kappa +1}\left(\frac{b}{\sqrt{b^{2}+\left(\kappa +1\right)K_{s}g_{0}}}-1\right)<0$$

As this equation is always negative, the concentration of active G protein must decrease as the concentration of GEF is increased (and vice-versa).

### Active G protein as a function of GEF concentration (with GTPase activity)

The effect of increasing the concentration of GEF on the steady-state concentration of active G protein with GTPase activity (*f*_GTPase_ = *k*_ase_[*G*_GTP_]) was investigated.

At steady-state $\frac{d[G_{\text{GTP}}]}{dt}=0$ implies:

$$\left[G_{\text{GDP}}\right]=\frac{K_{2}y^{2}+\left(K_{0}+\kappa \hat{\kappa }e_{0}\right)\left[G_{\text{GTP}}\right]}{\hat{\kappa }e_{0}-K_{1}\left[G_{\text{GTP}}\right]}$$ (6)

where $\hat{\kappa }=\frac{k_{\text{fwd}}}{k_{\text{ase}}}$.

Again assuming that *e*_0_ ≪ *g*_0_, Eq 2 simplifies to *g*_0_ = [*G*_GDP_] + [*G*_GTP_], into which Eq 6 can be substituted to obtain:

$$0=\left(K_{2}-K_{1}\right){\left[G_{\text{GTP}}\right]}^{2}+2\hat{b}\left[G_{\text{GTP}}\right]-\hat{\kappa }g_{0}e_{0}$$

where $\hat{b}=\frac{1}{2}(K_{0}+K_{1}g_{0}+\left(\kappa +1\right)\hat{\kappa }e_{0})$.

This quadratic equation has one solution that lies in the region 0 ≤ [*G*_GTP_] ≤ *g*_0_:

$$\left[G_{\text{GTP}}\right]=\frac{1}{K_{2}-K_{1}}\left(-\hat{b}+\sqrt{{\hat{b}}^{2}+\left(K_{2}-K_{1}\right)\hat{\kappa }g_{0}e_{0}}\right)$$

Alternatively, the proportion of active G protein is:

$$\frac{\left[G_{\text{GTP}}\right]}{g_{0}}=\frac{1}{g_{0}\left(K_{2}-K_{1}\right)}\left(-\hat{b}+\sqrt{{\hat{b}}^{2}+\left(K_{2}-K_{1}\right)\hat{\kappa }g_{0}e_{0}}\right)$$ (7)

The relative strength of the total forward catalytic activity of the GEF to the GTPase activity is given by the value of $e_{0}\hat{\kappa }$—though we are restricted by the constraint *e*_0_ ≪ *g*_0_, we remain unrestrained in $\hat{\kappa }$.

## Supporting Information

S1 FigApplication of the framework of [35] to the mechanism for GEF-mediated exchange of guanine nucleotides on G proteins.**A** The graph on the enzyme complexes with complexes as vertices and edges representing reactions labelled by rates and partner species. **B** All possible directed spanning trees of the graph on the enzyme complexes. The red vertex denotes the root of each spanning tree. **C** The basis element, *ρ*, generated from the each spanning trees: the sum over each root vertex, of the products of the labels of each spanning tree. Every steady-state of the original system *X* = ([*E*], [*E* ⋅ *G*_GDP_], [*E* ⋅ *G*_GTP_], [*E* ⋅ *G*])^*T*^ is a solution to the equation *X* = *λρ* where *λ* is a constant. We manipulate this equation to obtain $X_{i}=\frac{\rho_{i}}{\sum_{i}\rho_{i}}\times \sum_{i}X_{i}$.(TIFF)

S1 Table Concentrations, kinetic parameters, and summary parameters used for Figs 2 and 3. (PDF)

S1 Simulations Jupyter notebook containing Python code to produce the simulations shown in Figs 2 and 3. (IPYNB)
