# Supplementary figures and images for: Activation of G Proteins by Guanine Nucleotide Exchange Factors Relies on GTPase Activity
Source: PLoS One. 2016 Mar 17;11(3):e0151861. doi: 10.1371/journal.pone.0151861 (PMC4795700; doi:10.1371/journal.pone.0151861)

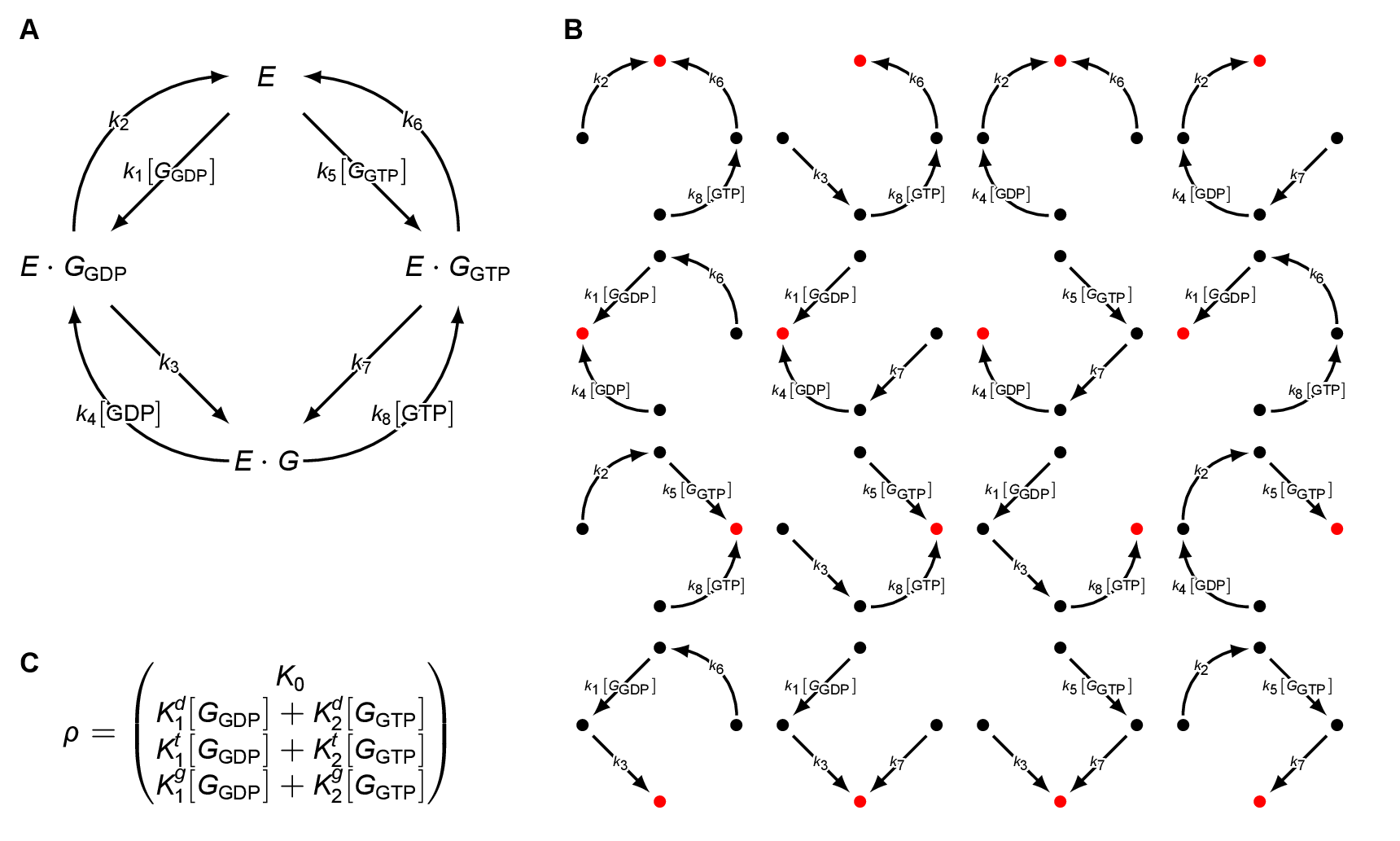

Supplement: S1 Fig — A The graph on the enzyme complexes with complexes as vertices and edges representing reactions labelled by rates and partner species. B All possible directed spanning trees of the graph on the enzyme complexes. The red vertex denotes the root of each spanning tree. C The basis element, ρ, generated from the each spanning trees: the sum over each root vertex, of the products of the labels of each spanning tree. Every steady-state of the original system X = ([E], [E ⋅ GGDP], [E ⋅ GGTP], [E ⋅ G])T is a solution to the equation X = λρ where λ is a constant. We manipulate this equation to obtain Xi=ρi∑iρi×∑iXi. (TIFF) [file pone.0151861.s001.tiff]
