# Supplementary material for: Activation of G Proteins by Guanine Nucleotide Exchange Factors Relies on GTPase Activity
Source: PLoS One. 2016 Mar 17;11(3):e0151861. doi: 10.1371/journal.pone.0151861 (PMC4795700; doi:10.1371/journal.pone.0151861)
Supplement: S1 Table — (PDF) [file pone.0151861.s002.pdf]

Parameter	Definition/source	Rever. model	Irrev. model	Units
e_0	916 ppm *	1.0 *(stimulated)*	1.0 *(stimulated)*	µM
f_0	202 ppm *	0.57	0.57	µM
g_0	114 ppm *	4.6	4.6	µM
[GTP]	Traut (1994)	305	305	µM
[GDP]	Traut (1994)	36	36	µM
k_1	Klebe, Prinz, et al. (1995)	74	74	$\mu M^{-1}\,s^{-1}$
k_2	Klebe, Prinz, et al. (1995)	55	55	s^{-1}
k_3	Klebe, Prinz, et al. (1995)	21	21	s^{-1}
k_4	Klebe, Prinz, et al. (1995)	11	11	$\mu M^{-1}\,s^{-1}$
k_5	Klebe, Prinz, et al. (1995)	100	100	$\mu M^{-1}\,s^{-1}$
k_6	Klebe, Prinz, et al. (1995)	55	55	s^{-1}
k_7	Klebe, Prinz, et al. (1995)	19	0	s^{-1}
k_8	Klebe, Prinz, et al. (1995)	0.6	0.6	$\mu M^{-1}\,s^{-1}$
k_{ase}	Klebe, Bischoff, et al. (1995)	2.1	2.1	s^{-1}
K_m	Klebe, Bischoff, et al. (1995)	0.43	0.43	µM
K_1^d	$k_1(k_6k_8[\mathrm{GTP}] + k_4(k_6+k_7)[\mathrm{GDP}])$	2.91×10^6	2.36×10^6	$\mu M^{-1}\,s^{-3}$
K_2^d	$k_4k_5k_7[\mathrm{GDP}]$	7.52×10^5	0	$\mu M^{-1}\,s^{-3}$
K_1^g	$k_1k_3(k_6+k_7)$	1.15×10^5	8.55×10^4	$\mu M^{-1}\,s^{-3}$
K_2^g	$k_5k_7(k_2+k_3)$	1.44×10^5	0	$\mu M^{-1}\,s^{-3}$
K_1^t	$k_1k_3k_8[\mathrm{GTP}]$	2.84×10^5	2.84×10^5	$\mu M^{-1}\,s^{-3}$
K_2^t	$k_5(k_8(k_2+k_3)[\mathrm{GTP}] + k_2k_4[\mathrm{GDP}])$	3.57×10^6	3.57×10^6	$\mu M^{-1}\,s^{-3}$
K_0	$k_6k_8(k_2+k_3)[\mathrm{GTP}] + k_2k_4(k_6+k_7)[\mathrm{GDP}]$	2.38×10^6	1.96×10^6	s^{-3}
K_1	$K_1^d + K_1^g + K_1^t$	3.31×10^6	2.73×10^6	$\mu M^{-1}\,s^{-3}$
K_2	$K_2^d + K_2^g + K_2^t$	4.47×10^6	3.57×10^6	$\mu M^{-1}\,s^{-3}$
k_{fwd}	$k_1k_3k_6k_8[\mathrm{GTP}]$	1.56×10^7	1.56×10^7	$\mu M^{-1}\,s^{-4}$
κ	$\frac{k_2k_4k_5k_7[\mathrm{GDP}]}{k_1k_3k_6k_8[\mathrm{GTP}]}$	2.65	0	—
K_s	$\frac{K_0}{(K_1\kappa+K_2)}$	0.18	0.55	µM

* Relative abundances were obtained from the 'Human, PaxDB integrated dataset' in the PaxDB database (Wang et al. 2012). An estimate of 3×10^6 proteins per μm^3 is given by Milo (2013). This gives an estimated total protein concentration of $\frac{3\times10^{24}}{6.022\times10^{23}} \approx 5\,\mathrm{mM}$ (1 ppm = 5 nM).

Klebe, C, F R Bischoff, H Ponstingl, and A Wittinghofer. 1995. "Interaction of the nuclear GTP-binding protein Ran with its regulatory proteins RCC1 and RanGAP1." *Biochemistry* 34 (2): 639–47.

Klebe, C, H Prinz, A Wittinghofer, and R S Goody. 1995. "The kinetic mechanism of Ran–nucleotide exchange catalyzed by RCC1." *Biochemistry* 34 (39): 12543–52.

Milo, Ron. 2013. "What is the total number of protein molecules per cell volume? A call to rethink some published values." *Bioessays* 35 (12): 1050–5.

Traut, Thomas W. 1994. "Physiological concentrations of purines and pyrimidines." *Mol. Cell. Biochem.* 140 (1): 1–22.

Wang, M, M Weiss, M Simonovic, G Haertinger, S P Schrimpf, M O Hengartner, and C von Mering. 2012. "PaxDb, a database of protein abundance averages across all three domains of life." *Mol. Cell Proteomics* 11 (8): 492–500.
